# Intracerebral Hemorrhage in a Patient with Untreated Rheumatoid Arthritis: Case Report and Literature Review

**DOI:** 10.7759/cureus.5175

**Published:** 2019-07-19

**Authors:** Omar Kousa, Dana H Awad, Yousif M Hydoub, Rasha Awawdeh, Venkata Andukuri

**Affiliations:** 1 Internal Medicine, Creighton University Medical Center, Omaha, USA; 2 Internal Medicine, Al-Mafraq Hospital, Abu Dhabi, ARE; 3 Internal Medicine, Al-Mafraq Hospital, Dubai, ARE

**Keywords:** rheumatoid arthritis (ra), intracerebral hemorrhage (ich), rheumatoid vasculitis(rv), hypertensive emergency

## Abstract

Rheumatoid vasculitis (RV) occurs in patients with long-standing rheumatoid arthritis (RA) or high levels of immunological factors and can result in devastating cardiovascular (CV) events. Here we report a case of a 38-year-old male who presented with hypertensive emergency and intracerebral hemorrhage (ICH). In the literature, a few observational studies have indicated the association of RA with hypertension; however, little evidence exists supporting a direct relationship between RA and ICH. In this case, we attempted to evaluate the complex relationship between all of these factors and found that early detection and treatment of RA may be beneficial in reducing ICH; however, large studies in the future are warranted to validate our observation.

## Introduction

Rheumatoid arthritis (RA) is a common, multisystem, autoimmune inflammatory disease. Despite the complexity of the disease, cardiovascular (CV) mortality remains the most common cause of death [1]. Although rheumatoid vasculitis (RV) is a known entity, it usually occurs in the setting of long-standing RA or with high levels of anti-citrullinated protein (anti-CCP) antibodies and rheumatoid factor (RF) [2]. The pathogenesis of vasculitis involves several mechanisms, one of which is immune-complex deposition in the sub-endothelium of affected vessels, a mechanism that may be augmented in patients with high levels of RF. Vasculitis has consistently been shown to be a major CV risk factor and it has also been hypothesized that systemic inflammation augments the effect of traditional CV risk factors, such as hypertension (HTN), lipids diseases and smoking, thereby increasing the risk of CV mortality and morbidity [3, 4]. However, central nervous system vasculitis in RA is rare. The literature contains only a few case reports on intracranial arteritis presenting as intracerebral hemorrhage (ICH) [5]. We aim, with this case report, to discuss the complex relationship between RA, hypertension and ICH, suggesting that early detection and treatment of RA is beneficial in reducing this risk.

## Case presentation

**Figure 1:**
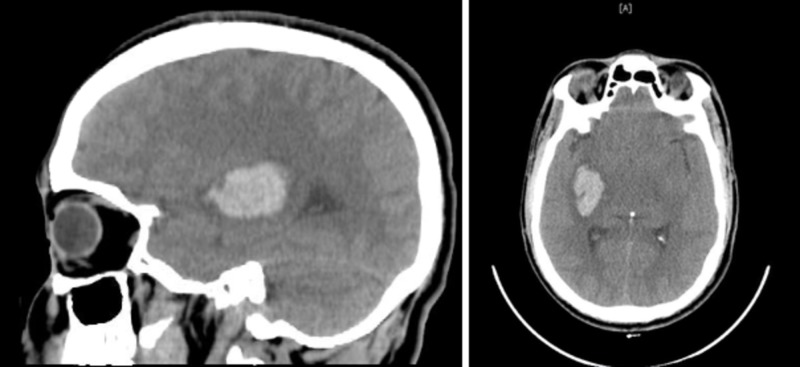
Computed tomography (CT) of the brain showing “a deep frontal lobe cerebral hemorrhage, mainly affecting the insula and involving the posterior limb of the internal capsule, with extrinsic compression of the ipsilateral thalamus.”

A 38-year-old male of Southeast Asian descent presented to the emergency department with a sudden onset of weakness of the left limb that lasted for four hours as well as a mild generalized headache with no alarming symptoms. He had no other symptoms of end-organ damage. He had been diagnosed with essential hypertension one year prior to presentation and was being treated using 25 mg of hydrochlorothiazide and was fairly controlled on that. Prior to admission, he did not suffer from any hypertensive retinopathy, nephropathy or hypertensive heart disease. His family history was unremarkable. On examination, his blood pressure (BP) was found to be elevated (160/110 mmHg) with no orthostatic changes. His other vital signs were within the normal range. An initial neurological examination revealed a muscle power of 3/5 in the left upper and lower limbs. He had 4+ deep tendon reflexes on the left side. The results of the remaining examination were normal. Funduscopic examination showed no papilledema or hypertensive retinopathy. Plain computed tomography (CT) of the brain (Figure 1) showed intra-cerebral hemorrhage. After a neurosurgical assessment, he was deemed suitable for non-operative conservative management, and he was admitted to the intensive care unit (ICU) and treated with intravenous labetalol. No further neurological deficit developed, and he was shifted to the general medical ward after 36 hours of monitoring in the ICU.

On further questioning, the patient complained of joints pain, mainly involving the metacarpophalangeal and proximal interphalangeal joints and wrists bilaterally, which started six months prior and had worsened over three weeks prior to admission. He also reported shoulder and knee arthralgia. He reported aching and stiffening of his hands, wrists, and shoulder joints that would be partially relieved after taking a hot shower. He had sought medical attention for the joint symptoms and was given nonsteroidal anti-inflammatory drugs, with no definitive diagnosis or indication for further evaluation and follow-up in the clinic.

An extensive examination of his joints showed tenderness and synovitis, bilaterally, including two to four of the proximal interphalangeal and metacarpophalangeal, wrists and shoulder joints. He had no skin manifestations or rheumatoid nodules.

Investigations

**Figure 2:**
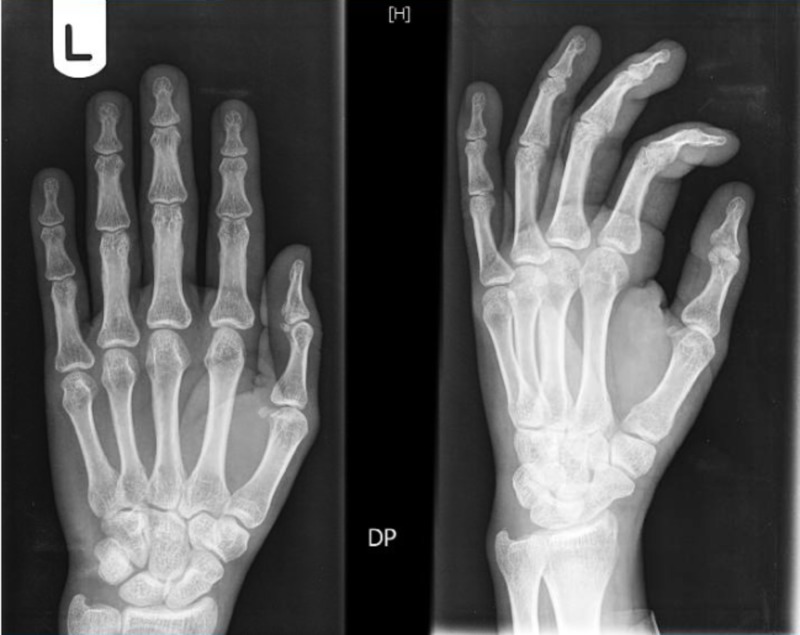
X-ray of the left hand showing “no bony abnormality, evidence of erosive arthropathy, or fracture or dislocation.”

**Figure 3:**
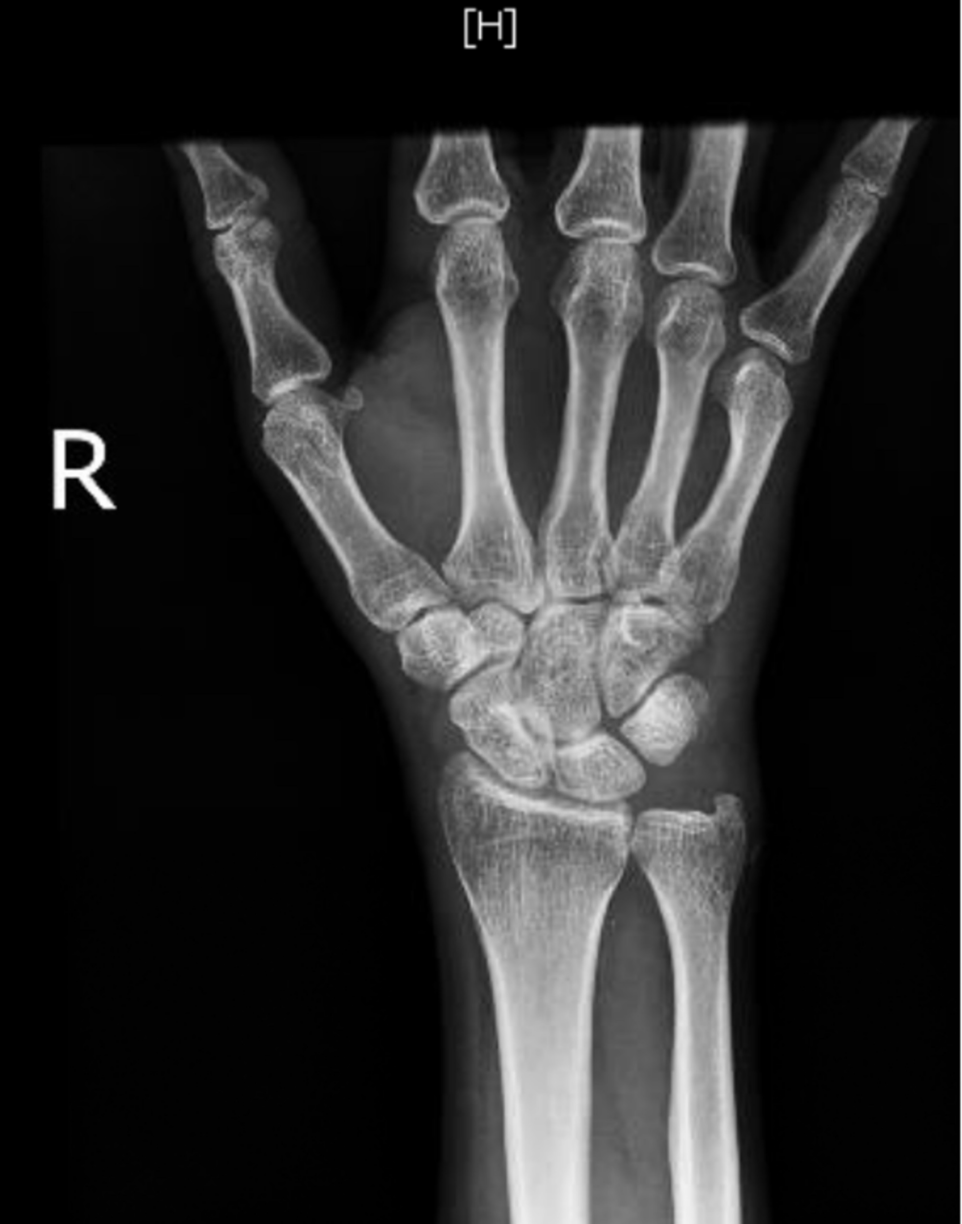
X-ray of the right hand showing “no bony abnormality, evidence of erosive arthropathy, or fracture or dislocation.”

**Figure 4:**
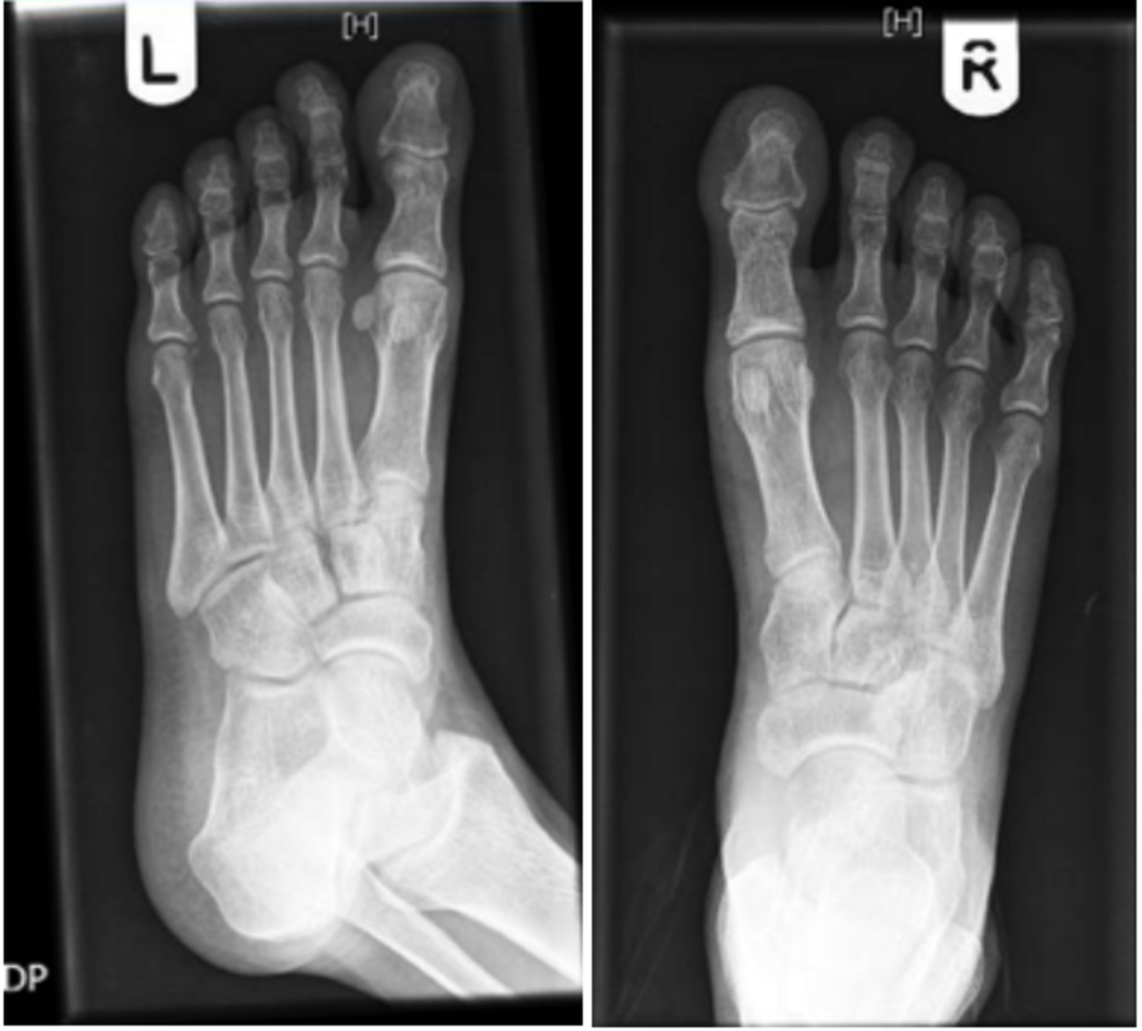
X-ray of the feet (the left foot and right foot are indicated by (L) and (R) on the top, respectively) showing “no bony abnormality, evidence of erosive arthropathy, or fracture or dislocation.”

**Figure 5:**
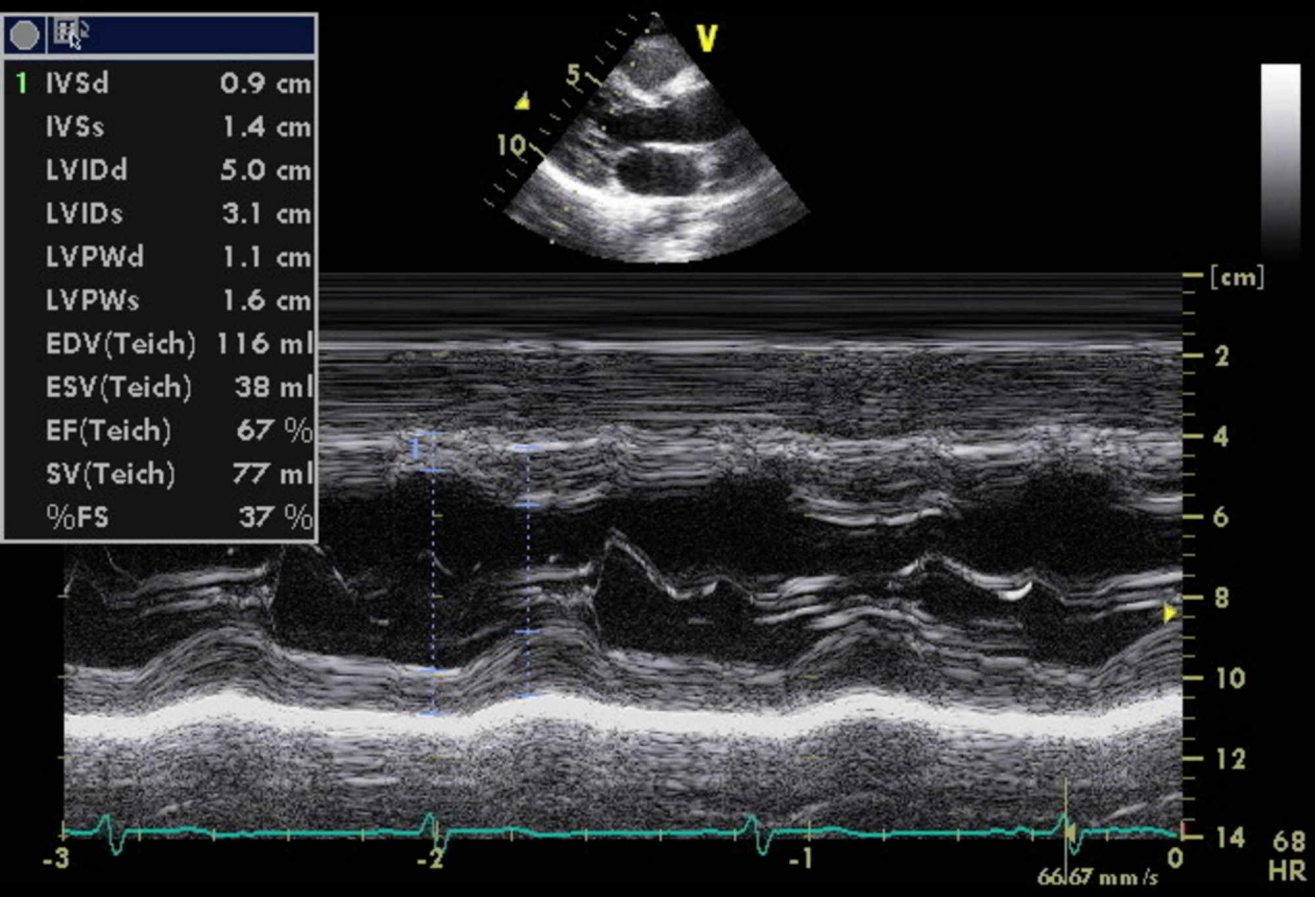
Trans-echocardiogram long axis view showing left ventricular thickness is slightly above 1.1 cm.

Laboratory investigations (Table 1) showed an elevated erythrocyte sedimentation rate and platelet count. The levels of RF and anti-CCP were significantly elevated (185 IU/ml and 117 units/ml, respectively). X-rays of hands and feet (Figures 2-4) showed no bony abnormalities. Workup for secondary causes of hypertension, including hyperaldosteronism, Cushing's disease, pheochromocytoma and renal artery stenosis, was negative. An electrocardiogram showed borderline left ventricular hypertrophy that was not present in a previous electrocardiogram obtained one year prior. A transthoracic echocardiogram showed normal systolic function and confirmed borderline left ventricular hypertrophy (Figure 5). A CT angiogram of the brain was performed, which revealed no sign of vasculitis.

**Table 1:** Laboratory results. ESR: Erythrocyte sedimentation rate; TSH: Thyroid-stimulating hormone.

	Result	Normal range
Sodium	141 mmol/l	135–145 mmol/l
Potassium	3.4 mmol/l	3.4–5.1 mmol/l
Chloride	101 mmol/l	98–107 mmol/l
Carbon dioxide	27 mmol/l	22–29 mmol/l
Creatinine	0.95 mg/dL	0.7–1.2 mg/dL
Urea	8.9 mg/dL	<23 mg/dL
Aldosterone standing	0.33 nmol/l	0.14–0.86 nmol/l
Renin standing	>128 nmol/l	4–37.52 nmol/l
Aldosterone supine	0.33 nmol/l	0.08–0.44 nmol/l
Renin supine	>128 nmol/l	4–23.7 nmol/l
Estimated glomerular filtration rate	87 ml/min/1.73 m^2^	> 60 ml/min/17.3 m^2^
Total urine volume	2.7 L	0.8–2.0 L
Urine metanephrine level every 24 h	1410 nmol/24 h/l	666-3691 nmol/24 h
White blood cell	9.81 × 10^9^/l	4–11 × 10^9^
Red blood cell	5.1 × 10^12^/l	4.3–5.7 × 10^9^
Hemoglobin	133 g/l	132–177 g/l
Hematocrit	0.424	0.34–0.49
Platelet	468 × 10^9^/l	140–450 × 10^9^
ESR	101, 80	1–13
Prothrombin time	13.4 s	11.5–14.5 sec
International normalized ratio	1.02	0.82–1.2
Activated prothrombin time	35.7 s	28.6–38.2 sec
DRVV screen	1.18	≤ 1.2
LUPUS INTERP	Negative	-
PTT-LA	40.1 s	29–45 s
Antinuclear antibody	Negative	
Cytoplasmic antineutrophil cytoplasmic antibodies	<2 RU/m	≤ 20 RU/m
Perinuclear anti-neutrophil cytoplasmic antibodies	<2 RU/m	≤ 20 RU/m
Complement 3	1.59 g/l	0.9–1.8 g/L
Complement 4	0.28 g/l	0.1–0.4 g/L
Cardiolipin IgG	3.7 units	≤ 10 units/mL
Cardiolipin IgM	1.00 units	≤ 7 units/mL
Cyclic citrullinated peptide antibodies	117 units/mL	≤ 5 units/mL
Anti-double-stranded DNA	<10.0 RU	≤ 100 RU/mL
Extractable nuclear antigens	Negative	-
Rheumatoid factor	185 IU/ml	<14 IU/ml
B2 glycoprotein immunoglobulin G	3 units/ml	<5 µg/ml
B2 glycoprotein immunoglobulin M	1 units/ml	<5 µg/ml
Low-density lipoprotein	135 mg/dL	0–100 mg/dL
High-density lipoprotein	20 mg/dL	35–77 mg/dL
Cholesterol	244 mg/dL	<205 mg/dL
Triglyceride	70 mg/dL	<90 mg/dL
Parathyroid hormone	3.1	1.6–6.9 pmol/l
Calcium	2.21	2.2–2.55 mmol/l
TSH	1.5	0.27–4.2 milli IU/l
Free tri-iodothyronine	4.2	3.1–6.8 pmol/l
Free Thyroxin	15	12–22 pmol/l
Early morning cortisol	12	10–22 µg/dl

Treatment

**Figure 6:**
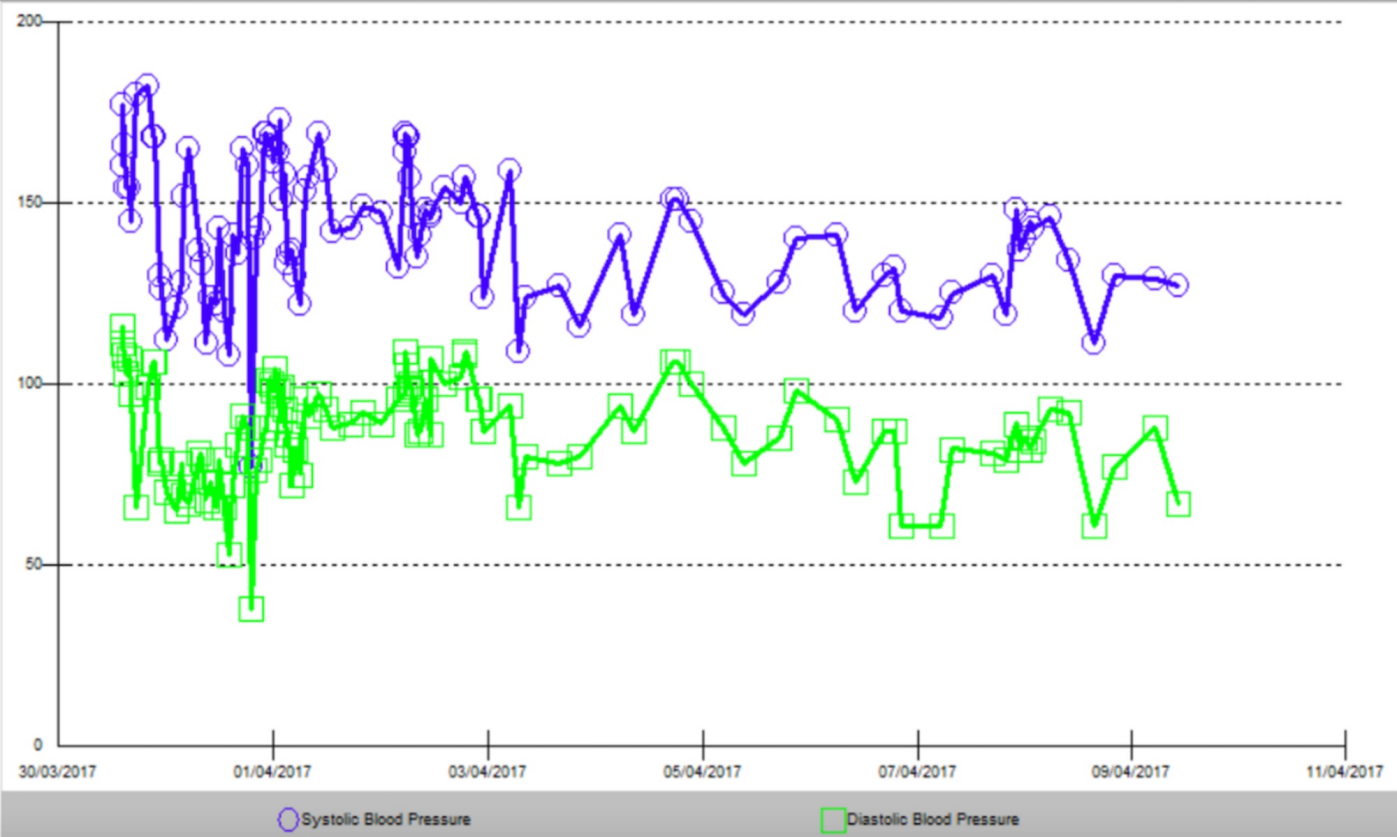
Systolic blood pressure and diastolic blood pressure.

During the first 24 hours, intravenous labetalol infusion was administered until a target mean arterial pressure of <110 mmHg was reached; subsequently, treatment was switched to intravenous boluses of labetalol (20 mg) to maintain the same target mean arterial pressure. After the first 24 hours, he was placed on oral amlodipine (10 mg) daily and metoprolol (50 mg) every 8 hours. Serial CT brain scans showed no progression of the hemorrhage; therefore, a neurosurgical intervention was not required. The diagnosis of RA was confirmed, and he was started on oral prednisolone (5 mg) daily and methotrexate (7.5 mg) weekly on the 5th day of admission. His BP started to improve after that (Figure 6). He was discharged home with close follow-up with a primary care physician and a rheumatologist. One month after discharge, his joint symptoms were controlled on methotrexate alone, and he was switched on ramipril 5 mg for his HTN, which fairly controlled his BP.

## Discussion

It is well established that RA increases the risk of CV events [1, 4]. After extensive efforts to quantify that risk, we now understand that the relationship is much more complicated than a simple cause and effect phenomenon. There has been a long-standing theory that RA causes intimal injury and arteriosclerosis through an intense inflammatory response [6]. Multiple cross-sectional and retrospective studies have demonstrated a higher prevalence of hypertension in patients with RA than in the general population [7]. Hence, it has become an interesting phenomenon that needs to be explained. Sesso et al. reported in his prospective observational study that C-reactive protein levels are associated with the future development of hypertension, in patients with inflammatory conditions [8]. However, there is little prospective evidence on a temporal relationship between RA, hypertension, and subsequent end-organ damage, including ICH.

Few cases of ICH in patients with RA have been reported in the literature [5]. Recently, two large population-based cohort studies associated various immunological diseases, including RA, with ICH and/or subarachnoid hemorrhage [3, 9]. Both studies have found that the risk of ICH is higher in RA patients, with the highest risk occurring during the first year of diagnosis of RA. However, both of these studies lacked any structured follow-up and they did not consider various important confounding factors such as body mass index, smoking, blood pressure, and race.

In a meta-analysis of 23 studies, it was found that patients with RA have a risk of hemorrhagic stroke 1.68 times higher than that in normal individuals [4]. The end-organ damage because of hypertension is considered to be more prevalent in patients with rheumatoid arthritis than in the general population [10]. Our patient had developed ICH, although there was no record of BP ever having been above 180/120 mmHg. It is thought that the risk of ICH exponentially increases when BP exceeds 180/120 mmHg. Hemorrhage rarely occurs when BP is below that range unless there is a sudden and rapid increase in BP [11]. Other than borderline left ventricular hypertrophy, our patient had no evidence of acute or chronic hypertensive end-organ damage.

The fact that the patient’s hypertension had been diagnosed a year prior to his stroke with no evidence of chronic hypertensive end-organ damage, had been controlled on a single oral medication with full compliance, and that a BP greater than 180/120 mmHg had never been recorded during his hospitalization, led us to look for another explanation for his susceptibility to ICH. He had no coagulopathies, all immunological markers for vasculitis tested negative, and CT angiogram did not reveal cerebral vasculitic lesions or other forms of gross vasculopathy.

Detailed questioning revealed that his six-month history of polyarthralgia was not properly investigated and was undiagnosed. The early subclinical joint inflammation and lack of other physical signs of RA, as well as the normal X-rays of his joints before hospital admission had likely prevented the diagnosis of early RA. He had active RA with high levels of inflammatory markers. Based on the American College of Rheumatology criteria, the diagnosis of RA was established.

The patient was started on oral prednisolone which dramatically controlled his joints symptoms. In addition, his BP could not be controlled with anti-hypertensives until after prednisolone was started on the fifth day of hospitalization (Figure 6).

It has been observed that controlling hypertension in patients with RA can be quite challenging compared with that in the general population. In a cross-sectional study, it was found that optimal control of hypertension in patients with RA was significantly lower at 13.2% than the 21%-23% observed in the general population [12]. Because inflammation is thought to be a contributing factor in this phenomenon it has been suggested that cardiovascular medications with anti-inflammatory properties, such as angiotensin-converting enzyme inhibitors, exhibit enhanced antihypertensive properties, particularly when administered to patients with RA [12].

Although hypertension is a long-term side effect of the chronic use of oral steroids used for treating RA [13]. It remains unclear to what degree can corticosteroids induce hypertension in patients with RA. It is interesting to note that hypertension in our patient was not perfectly controlled in the hospital until after the fifth day of admission when prednisolone was initiated (Figure 6). Although this may have been a coincidence, it might have played a role in controlling the inflammation thereby helping control resistant hypertension observed in patients with RA. This case gave us an opportunity to consider the complex role of inflammatory mediators in RA and their possible effects on BP and cardiovascular diseases.

## Conclusions

It is well known that RA increases the risk of thromboembolic disease, however, a relationship with ICH is not yet well established. If indeed a direct causative link can be made between the inflammatory state in patients with RA and an increased risk of hemorrhagic stroke, it will be interesting to know whether diagnosing and treating RA early in the disease would reduce this risk. It would require further investigations into the pathophysiology of such strokes with regard to inflammatory vasculitis as well as prospective studies on patients with RA to ascertain whether good disease control actually reduces the risk of ICH or any other cardiovascular events. If such an effect can be shown, then reduction of cardiovascular risk in such patients may well begin in the rheumatologist’s office.
